# Supplementary material for: Measuring global multi-scale place connectivity using geotagged social media data
Source: Sci Rep. 2021 Jul 19;11:14694. doi: 10.1038/s41598-021-94300-7 (PMC8290042; doi:10.1038/s41598-021-94300-7)
Supplement: Supplementary file 1 — Supplementary Information 1. [file 41598_2021_94300_MOESM1_ESM.pdf]

Measuring Global Multi-Scale Place Connectivity using Geotagged Social Media Data

Appendices

Zhenlong Li[1*], Xiao Huang[2], Xinyue Ye[3], Yuqin Jiang[1], Yago Martin[4], Huan Ning[1], Michael E. Hodgson[1], and Xiaoming Li[5]

[1] *Geoinformation and Big Data Research Laboratory, Department of Geography, University of South Carolina, SC, USA*

[2] *Department of Geosciences, University of Arkansas, AR, USA*

[3] *Department of Landscape Architecture & Urban Planning, Texas A&M University, TX, USA*

[4] *School of Public Administration, University of Central Florida, FL, USA*

[5] *Department of Health Promotion, Education, and Behavior, University of South Carolina, SC, USA*

[*] zhenlong@sc.edu

A. Directional (or Asymmetrical) PCI

The same number of shared users between two places may have a different impact on each place. Suppose that two places *i* and *j* have 50 shared users, place *i* has 100 users in total, and place *j* has 1000 users in total. In such a case, place *j* can be considered to have a larger impact on place *i* as it has 50% of users shared with place *j*. Similarly, place *i* has a smaller impact on place *j* as it only has 5% of users shared with place *i*.

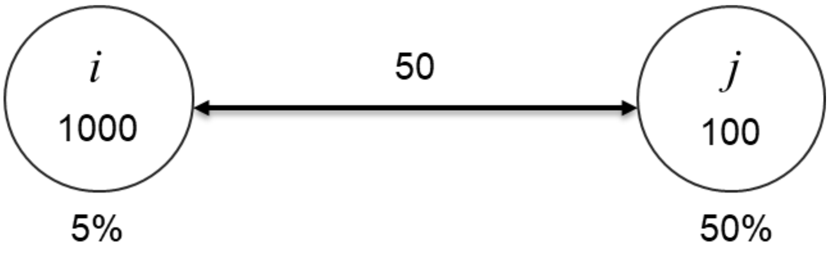

Fig. A1. Illustration of the asymmetrical impact of the shared users on different places

To capture the asymmetrical impact of the same number of shared users on different places, a directional PCI can be derived from Eq. A1 and Eq. A2.

$$PCI_{i \to j} = \frac{s_{ij}}{s_j} \qquad i, j \in [1, n] \qquad \text{Eq. A1}$$

$$PCI_{j\to i} = \frac{S_{ij}}{S_i} \qquad i,j \in [1,n] \qquad \text{Eq. A2}$$

Where $PCI_{i\to j}$ denotes the impact of place *i* on place *j*, and $PCI_{j\to i}$ denotes the impact of place *j* on place *i*. S_i is the number of observed persons (unique social media users) in place *i* within time period *T*; S_j is the number of observed persons in place *j* within time period *T*; S_{ij} is the number of shared persons between places *i* and *j* within time period *T;* and *n* is the number of places in the study area. For the two places illustrated in Figure A1, the directional $PCI_{i\to j}$ = 0.500, $PCI_{j\to i}$ = 0.050, and the non-directional PCI_{ij} = 0.158.

Same as the PCI (non-directional or symmetrical) discussed in the paper, the directional (or asymmetrical) PCI was also computed for the following four geographic levels: 1) 2019 census tract level PCI for the Las Angeles county, US, 2) 2018 and 2019 county-level PCI for the entire contiguous US, 2) 2019 worldwide first-level subdivision PCI, and 4) 2019 worldwide country level PCI. While the directional PCI makes reasonable sense conceptually, understanding its characteristics and applications requires further investigation.

To facility further studies, the derived asymmetrical PCI can be downloaded at https://data.humdata.org/dataset/social-connectedness-index, and be visualized/explored at http://gis.cas.sc.edu/GeoAnalytics/pci.html.

B. Descriptive Statistics of Worldwide Geotagged Tweets in 2019

We collected 1,437,611,832 worldwide geotagged tweets in 2019 using the public Twitter Streaming Application Programming Interface (API). Following Martin et al. (2020), we filtered out the non-human tweets (e.g., automated weather reports, job offers, and advertising) by checking the tweet source from which application a tweet is posted. For example, tweets automatically posted for job offers from the source TweetMyJOBS and CareerArc are removed. A list of the Twitter sources that indicate human-posted tweets selected by manual checking is shown in Table B1. Only tweets posted from these sources were included in the analysis and PCI computation. After filtering, a total of 1,409,404,996 geotagged tweets posted by 17,013,612 unique Twitter users were selected. Fig. B1 shows the spatial distribution of these filtered tweets at the country level. The top 40 countries with the most observed unique Twitter users in 2019 are reported in Table B2. Fig. B2, B3 show the number of unique Twitter users observed in 2019 at the county level in the US and census tract level in NYC.

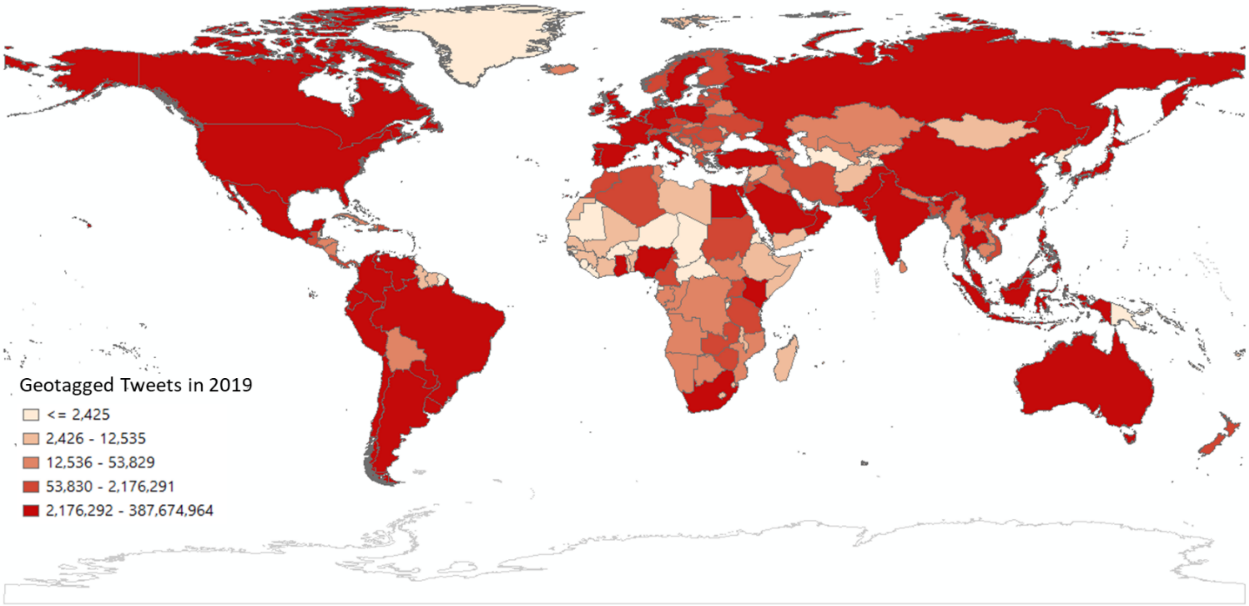

Fig. B1. Spatial distribution of the geotagged tweets at the country level in 2019 (with non-human tweets removed)

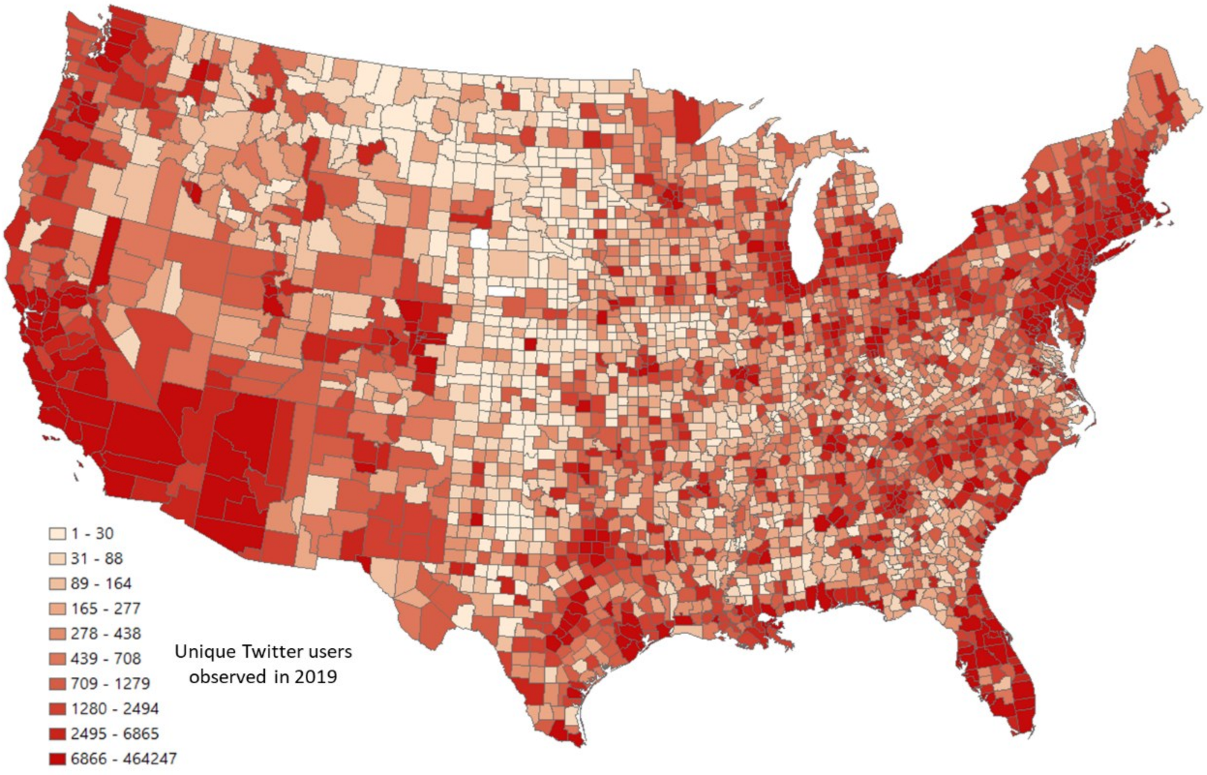

Fig. B2. Spatial distribution of the observed unique Twitter users at the county level in 2019 (with non-human tweets removed). (Mean number of users per county: 3822, Median number of users per county: 431 users, County count: 3108)

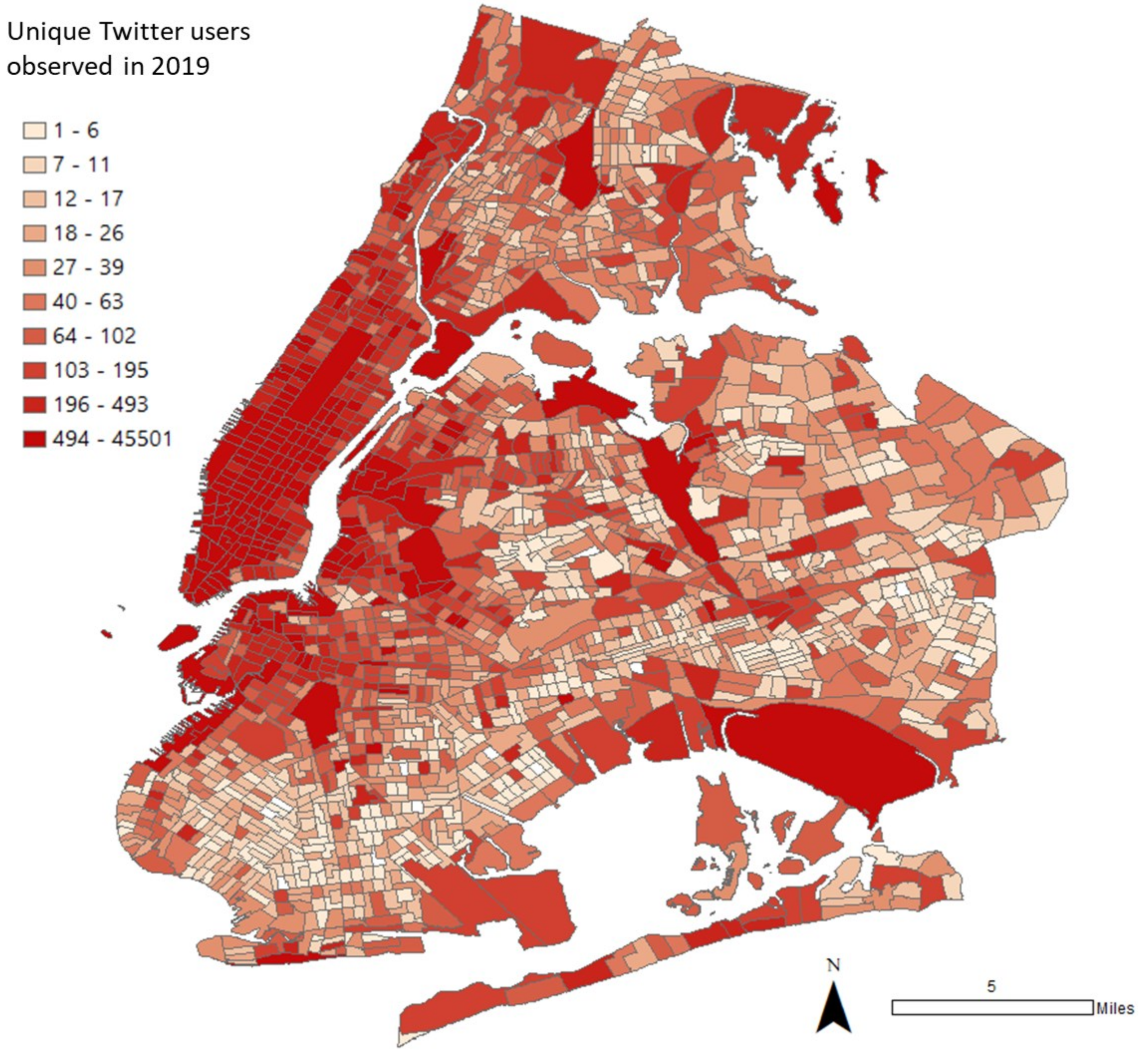

Fig. B3. Spatial distribution of the observed unique Twitter users based on only the coordinates, neighborhood, and point of interest tweets at the census tract level in New York City in 2019 (with non-human tweets removed). (Mean number of users per county: 324, Median number of users per county: 37 users, Tract count: 2056)

Table B1. List of Twitter sources that indicate human-posted tweets selected by manual checking.

TweetCaster for Android, TweetCaster for iOS, Tweetings for iPad, Tweetings for Android, Tweetings for Android Holo, Tweetings for Android Tablets, Tweetings for iPhone, Tweetings for iPhone, Tweetlogix, twicca, Twidere for Android #4, Twidere for Android #5, Twidere for Android #7, Twishort Client, Twittelator, Twitter Dashboard for iPhone, Twitter Engage for iPhone, Twitter for Android, Twitter for iPhone, Twitter for Android, Twitter for Android Tablets, Twitter for Apple Watch, Twitter for BlackBerry, Twitter for Calendar, Twitter for iPad, Twitter for iPhone, Twitter for Mac, Twitter for Windows, Twitter for Windows Phone, Untappd, Tweetbot for iOS, Foursquare Swarm, UberSocial for Android, Twitter Web Client, Gay Los Angeles, Gay Santa Monica, Gay West Hollywood, Hootsuite, Instagram, iOS, OS X,

PlumeforAndroid, SoundHound,Squarespace, Talon (Plus), Talon Android, Talon Plus, Echofon, Endomondo, Fenix for Android, Flamingo for Android, Foursquare, Tweet It! for Windows, Tweetbot for iS, Tweetbot for Mac

Table B2. Top 40 countries with most observed unique Twitter users in 2019

ISO_Code	Users	Tweets
USA	4704692	387674964
BRA	1284039	215233195
GBR	1172344	70927892
JPN	1087206	84795389
IND	869366	33546715
IDN	797242	40052233
TUR	717647	32230682
ESP	612306	31554779
MEX	594386	29117892
PHL	571031	60788557
SAU	517797	30535954
FRA	487938	21611155
ARG	410228	40673385
CAN	384644	19893446
THA	349012	16894003
ITA	302492	12711959
MYS	300914	23034466
DEU	273307	9885420
COL	236920	14753358
NGA	218728	20002082
NLD	191777	7154017
ZAF	180112	17589164
AUS	179728	9860744
ARE	172797	6222886
EGY	156265	9962841
CHN	150889	2417144
CHL	143661	10082759
RUS	136230	10002772
PRT	114156	6391299
KOR	111488	4700062

The locations embedded in the geotagged tweets have different spatial resolutions (e.g., exact coordinates, neighborhood, city, and country, etc.). We also analyzed the locations of the 1.4 billion geotagged tweets as the spatial resolution of a geotagged tweet needs to be considered when computing PCIs at different geographic levels. For example, for computing PCI

at the US county level, we need to filter out tweets geotagged at a geographic level lower than a city (state level and country level tweets were excluded). As shown in Fig. B4, a majority of tweets (1.1 billion, 79%) were geotagged at the city level, followed by first-level subdivision such as state or province (138.1 million, 9.8%), exact coordinates, (90.4 million, 6.4%), country level (46.2 million, 3.3%), and neighborhood/point of interest (POI) (21.4 million, 1.5%). Based on this, 86.9% (i.e., city-level, exact coordinates, and neighborhood) of the geotagged tweets can be used for computing the inter-city (county) level PCI. 96.8% of tweets can be used for the first-level subdivision level PCI, and all geotagged tweets can be used for computing country level PCI. For intra-city level PCI (e.g., US census tract level), tweets at the coordinates, neighborhood, or POI levels can be used. This indicates that the removal of precise geotagging function by Twitter has little impact on the computation of PCI at the county level or above (e.g., world first-level admin and country).

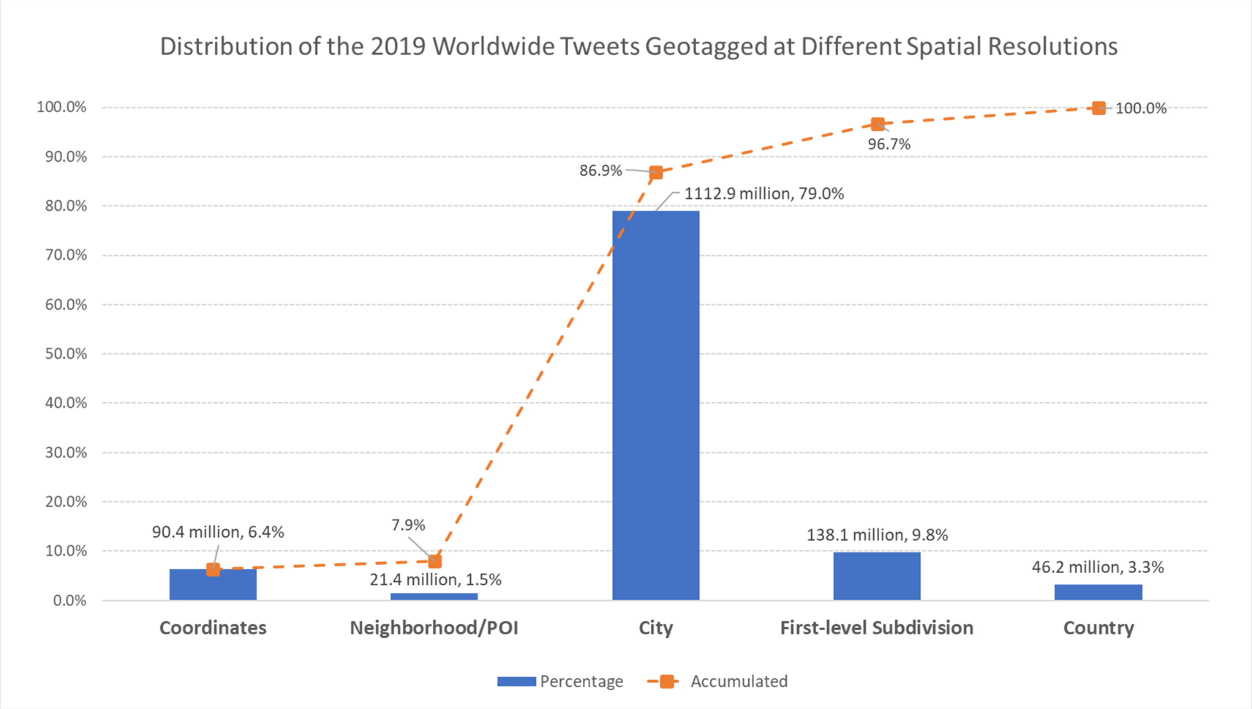

Fig. B4. Distribution of the 2019 worldwide tweets geotagged at different spatial resolutions.

C. Computation of US County Level PCI Using 2019 Geotagged Twitter Data

A total of 391,503,203 geotagged tweets from 4,892,458 distinct Twitter users were extracted within the bounding box covering the contiguous United States. For computing PCI at the county level, we filtered out tweets geotagged at a geographic level lower than a city (e.g., state level). If a tweet is geotagged at the place level, the coordinates (latitude and longitude) of the place centroid were used in the analysis. We filtered out the non-human tweets following the procedure introduced in Appendix B. After spatial filtering and non-human filtering, a total of 316,797,441 tweets remained from 4,609,040 unique Twitter users. The process was performed using Apache Impala in a Hadoop environment.

After data cleaning, each Twitter user was assigned to a county based on that user's tweet location daily. For example, if a user tweeted in three counties on a specific day, then for that user on that day, three rows were generated. After processing all users, counties, and days in 2019, a big table (*CountyUserDate*) with three fields (*county, user, date) was generated,* including 95,701,425 county-user-date tuples. The daily table was then aggregated along the date to produce a new table (*CountyUserDays*) with three fields (*county, user, days*), where *days* indicates the number of days (in 2019) a user was observed in a county, including 11,875,433 county-user-days tuples. Based on the *CountyUserDays* table, for each county, two numbers were derived: 1) the number of shared users with other counties and 2) the number of total observed users in each county. Finally, PCI was computed for each county pair (those with shared users) using Eq. 1. This process was conducted using Apache Hive coupled with Esri GIS tools for Hadoop (Esri, 2019).

D. Computation of the County Person-Day Movements Using 2019 SafeGraph Data

The SafeGraph's Social Distancing Metrics (SDM) data (SafeGraph, 2020) were downloaded and loaded to our Hadoop cluster. There were 23 fields in the SDM table, and three were used to derive the population movement, including *origin_census_block_group*, *destination_cbgs*, and *date_range_start*. The *origin_census_block_group* is the unique 12-digit FIPS code for the Census Block Group. *Destination cbgs* contains a list of key-value pairs with key indicating the destination census block group (from the origin census block group) and "value is the number of devices with a home in *census_block_group* that stopped in the given destination census block group for >1 minute during the time period" (https://docs.safegraph.com/docs/social-distancing-metrics). The *date_range_start* was used to extract the date information.

Based on the three fields, we generated a new table (*SgDailyOD*) with each row showing the number of devices from an original block group to a destination block group on a specific day, resulting in over 6 billion (6,144,802,397) origin-destination flows. Based on the *SgDailyOD* table, we further aggregated the flows to the county level for 2019, generating a new table (*SgCountyPersonDayMovement*) with each row showing the total number of device movements between two counties on all days of 2019, resulting in over 6 million county pairs (6,119,765). Note that the number of movements includes the movements from both directions. For instance, if there are *m* number of movements from county A to county B, and *n* number of movements from county B to county A, then the number of movements between the two counties is calculated as $m + n$. The entire process was conducted in our in-house Hadoop cluster using Apache Hive and Impala.

E. Computation of the County Person-Day Movements Using 2019 Geotagged Twitter Data

After data cleaning, each Twitter user was assigned to a county based on that user's tweet location daily. For example, if a user tweeted in three counties on a specific day, then for

that user on that day, three rows were generated. After processing all users, counties, and days in 2019, a big table (*CountyUserDate*) with three fields (county, user, date) was generated, including 95,701,425 county-user-date tuples. This step was conducted using Apache Hive coupled with ESRI tools for Hadoop. The person-day movement with geotagged tweets was calculated by aggregating the *CountyUserDate* table along the date, generating a new table (*SgCountyPersonDayMovement)* with each row showing the total number of user movements between two counties on all days of 2019, resulting in over 3 million (3,405,113) county pairs.

F. Person-day movement distribution histogram for two selected counties to other counties illustrating the highly skewed nature of the data.

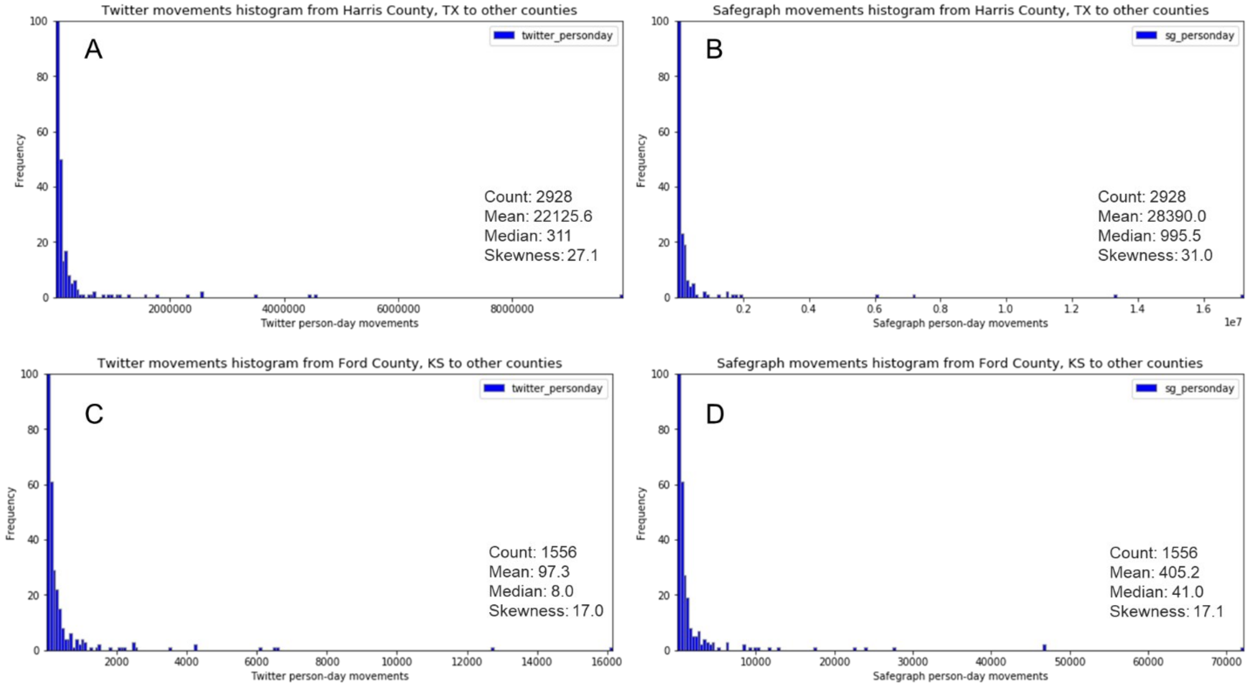

Figure F. Histogram of (A). Twitter-derived person-day movements from Harris County, TX to other counties, (B). SafeGraph-derived person-day movements from Harris County, TX to other counties, (C). Twitter-derived person-day movements from Ford County, KS to other counties, (D). SafeGraph-derived person-day movements from Ford County, KS to other counties. (Note that Y axis for each histogram is cut off at 100 for the display purpose). The skewness values are large positive, and the mean and median are dramatically different for each example.

G. Results of the hierarchical agglomerative clustering of 2019 PCI at the worldwide country first-level subdivision level with 50 and 200 targeted numbers of communities.

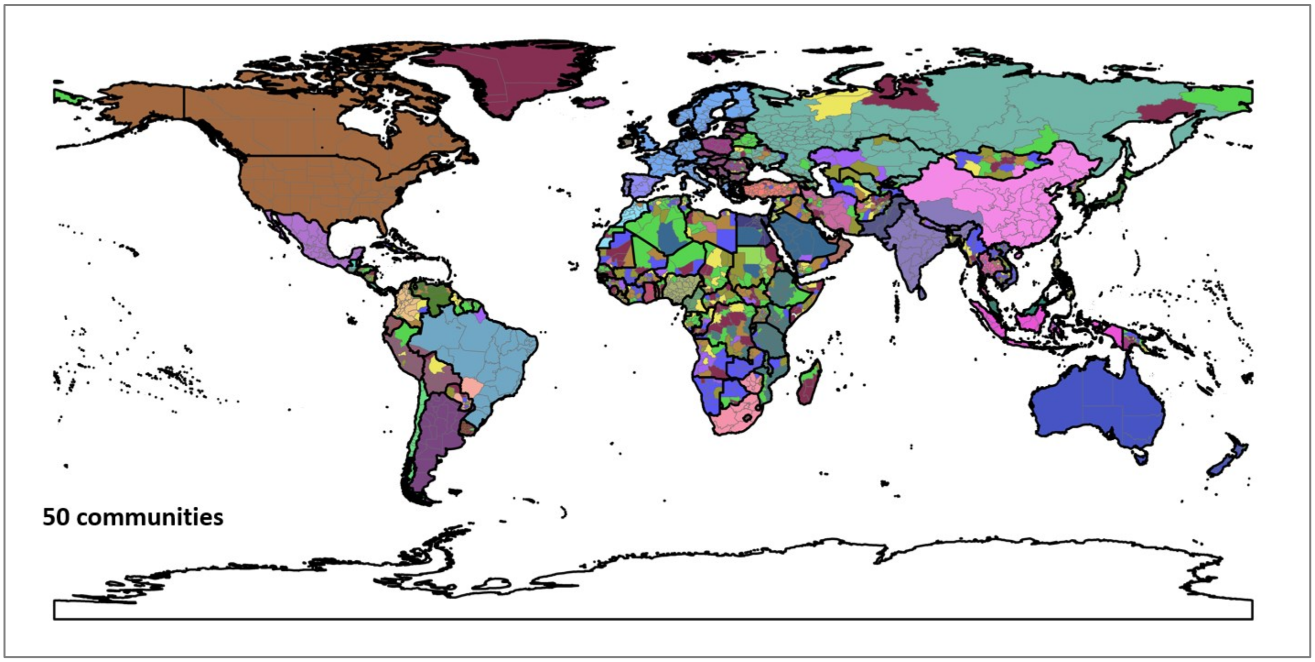

Fig. G1. Clustering result of 50 communities. Each color depicts a community. Boundary data was retrieved from GADM (2018).

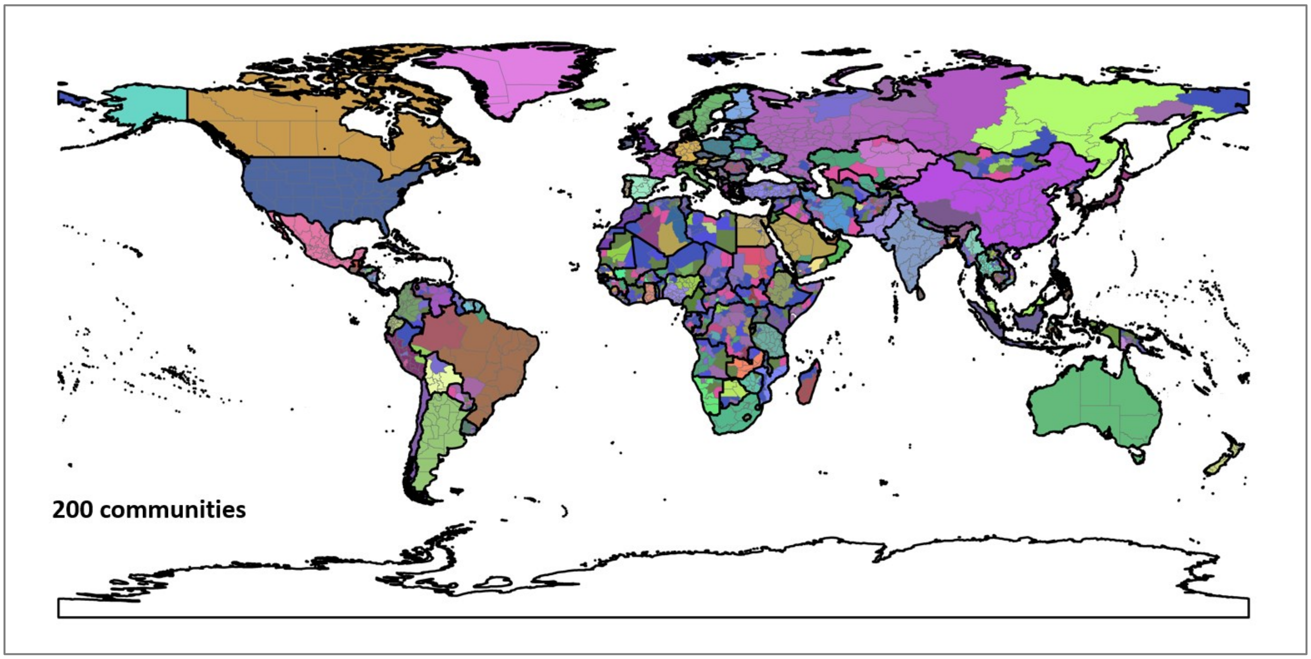

Fig. G2. Clustering result of 200 communities. Each color depicts a community. Boundary data was retrieved from GADM (2018).

References

Martin, Y., Cutter, S. L., Li, Z., Emrich, C. T., & Mitchell, J. T. (2020). Using geotagged tweets to track population movements to and from Puerto Rico after Hurricane Maria. Population and Environment, 42(1), 4–27.

ESRI. (2019). GIS Tools for Hadoop: Big data spatial analytics for the Hadoop framework. https://esri.github.io/gis-tools-for-hadoop/

SafeGraph. (2020). Social Distancing Metrics. SafeGraph. https://docs.safegraph.com/docs/social-distancing-metrics

GADM, (2018), GADM data at https://gadm.org/data.html, last accessed on January 5, 2021
